# Epilepsy and the cortical vestibular system: tales of dizziness and recent concepts

**DOI:** 10.3389/fnint.2013.00073

**Published:** 2013-11-11

**Authors:** Russell Hewett, Fabrice Bartolomei

**Affiliations:** ^1^Department of Neurology and Neurophysiology, Institute of Neurological Sciences, Southern General HospitalGlasgow, UK; ^2^Clinical Neurophysiology and Epileptology Department, Hôpital de la TimoneMarseille, France

**Keywords:** epilepsy, vertigo, vestibular system, EEG, cerebral cortex

## Abstract

Cortical representations of the vestibular system are now well recognized. In contrast, the fact that epilepsy can affect these systems, provoking transient vestibular symptoms, is less known. Focal seizures may nonetheless manifest by prominent vestibular changes ranging from mild unsteadiness to true rotational vertigo. Most often these symptoms are associated with other subjective manifestations. In pure vestibular forms, the diagnosis may be more difficult and is often delayed. The cortical origin of these symptoms will be discussed and compared with the known “vestibular” cortical representations. In addition, the existence of a specific “vestibular epilepsy” has been suggested in some publications. This condition affects young subjects with a frequent family history and most often a benign evolution, raising the possibility of a form of idiopathic epilepsy (Hewett etal., 2011).

## INTRODUCTION

The vestibular symptoms of vertigo and disequilibrium are frequent subjective symptoms with a wide spectrum of peripheral and central causes (Neuhauser et al., 2005, 2008). Although epilepsy has been historically linked with vertigo (Gowers, 1907; Alpers, 1960; Smith, 1960; Gordon, 1999), albeit for misunderstood reasons, it has more recently been considered an extremely rare cause and often ignored by clinicians and neurologists (Brandt, 2003; Dieterich, 2007; Dieterich and Brandt, 2008).

However, as the evidence for the cortical representation of the vestibular system grows (Penfield, 1957; Guldin and Grusser, 1998; Brandt and Dieterich, 1999; Blanke et al., 2000; Duque-Parra, 2004; Best et al., 2010; Lopez and Blanke, 2011; Lopez et al., 2012), so does the evidence for vestibular symptoms occurring as a manifestation of associated focal epileptic activity (Penfield and Kristiansen, 1951; Penfield and Jasper, 1954; Kogeorgos et al., 1981; Kahane et al., 2003; Hewett et al., 2011).

Here we discuss the changing perception of the role of seizure activity in producing vestibular symptoms, and review the recent evidence that describe a pure vestibular epilepsy and raise the possibility of a idiopathic vestibular epileptic syndrome (Hewett et al., 2011).

## HISTORICAL REVIEW

### VERTIGO AND EPILEPSY

From ancient times the term vertigo and epilepsy have been linked conceptually and diagnostically. Bladin’s excellent historical review of “epileptic vertigo” documents that in second century A.D. Arataeus stated that if vertigo proved incurable, it might be the beginning of chronic epilepsy (Bladin, 1998) whilst vertigo was also reported to be considered “a little epilepsy” (Temkin, 1994).

However, the term vertigo had a much looser definition than the illusion of surrounding or self-motion understood in present times and was considered more a manifestation of a non-specific paroxysmal cerebral disturbance (Gizzy and Diamond, 2005). It was only when analysis of the sense of motion began in the late 19th century (Baloh, 2001) did the modern understanding of vertigo as a predominantly otogenic symptom arising from the vestibular system take hold.

Vertigo and epilepsy was first formally linked and consolidated into the term “epileptic vertigo” during the pioneering years of the scientific study of epilepsy in 18th and 19th century by French clinicians. Esquirol in 1838 first introduced the concept as a grade of epileptic severity that included “vertige epileptique,” “petit mal” and “grand mal” (cited in Temkin, 1994) and even with the introduction of absences to describe minor epileptic episodes, the term persisted.

Generalized seizures were considered to originate from the upper brainstem, but the condition of epileptic vertigo was thought to be localized to the hemispheres and to play a role in the mental symptoms of epilepsy. This poorly defined clinical entity developed negative connotations after research from the asylums of Paris associated aberrant behavioral episodes of the patients with brief incomplete epileptic attacks. Epileptic vertigo and thus epilepsy soon became popularly linked with the potential to suffer acute attacks of wayward potentially violent behavior (review in Bladin, 1998). The term remained in general use until the early 20th century and was accepted by many of the prolific authors of the time (Gizzy and Diamond, 2005), including Hughling Jackson:

“I believe that epileptic vertigo, epileptic petit-mal, and epileptic grand-mal are when regarded from an anatomical and physiological point of view simply differing degrees, that is to say they depend on different strengths of discharge...” (Hughlings Jackson, 1931).

However, the definition of the condition continued to lack of clarity leading to considerable disagreement (Bladin, 1998).

### VERTIGO: EPILEPSY VERSUS THE VESTIBULAR SYSTEM

In 1861 Meniere’s localisation of vertigo to the inner ear drove the conceptual separation of vertigo from epilepsy (Baloh, 2001), though the shift did not occur immediately.

By the 1870’s Charcot, Jackson and Gowers had recanted their early concepts and strongly accepted that most vertigo emanated from the inner ear (Gordon, 1999).

In the Borderlands of Epilepsy 1907 (Gowers, 1907), Gowers remarks on the former imprecise descriptions of vertigo: “The sense in which it is popularly used is very wide and includes every peculiar vague brain sensation, especially brief obscuration of consciousness, imperfect perception of surroundings and the like.”

By the end of the 19th century, most authors had assigned vertigo to conditions other than epilepsy. If associated with epilepsy, vertigo was considered a symptom that may be felt at the onset of a seizure and even if it accompanied epilepsy, it conferred nothing of significance regarding classification or degree of severity (Bladin, 1998).

However, Gowers, being a master of medical observers, was probably the only great author in the late 19th century to still be able to consider it as a possible epileptic symptom and made great effort in making the distinction between the two entities.

“The attacks of minor epilepsy which are characterized by vertigo have to be distinguished from the other form of sudden giddiness” (Gowers, 1906).

### 20th CENTURY AND VESTIBULAR EPILEPSY

The term epileptic vertigo did not disappear from the literature. It continues along with epileptic dizziness, epileptic nystagmus and epileptic tinnitus as conditions purely describing the predominant symptom associated with a seizure or epilepsy (Esquirol cited in Temkin, 1994).

As the field of epileptology advanced with the widespread acceptance of a cortical basis to seizure genesis, the 20th century saw more directed effort to understand the cortical substrate for the production of vertigo and vestibular disturbance associated with focal epileptic activity.

The electrical stimulation studies in humans and monkeys in the early part of the century gave the first insight to anatomical basis of the vestibular cortex whereas more recently advances have been made by the combination of intracranial stimulation studies in medically intractable epilepsy patients and modern electroencephalography (EEG) techniques, structural and functional imaging (reviewed in Lopez and Blanke, 2011).

## THE VESTIBULAR CORTEX IN EPILEPSY

In comparison to the wealth of data collected with regards to the visual and auditory cortices, less is known of the vestibular cortical representation and the processing of vestibular information. Data from tracer and electrophysiological studies on non-human primates have demonstrated multiple distinct vestibular cortical areas (Guldin and Grusser, 1998) with a parieto-insular vestibular cortex (PIVC) as a proposed core vestibular region. This can be directly been compared to clinical work and neuroimaging in humans (Lopez and Blanke, 2011), as well as recent meta-analyses of neuroimaging studies that more specifically propose the parietal operculum and posterior insula as candidates for the primary vestibular cortex (Lopez et al., 2012; zu Eulenburg et al., 2012).

In particular focal brain stimulations in epileptic patients have added to the mounting evidence of human vestibular cortical representation.

### STIMULATION STUDIES IN EPILEPTIC PATIENTS AND VESTIBULAR SYMPTOMS

During electrical cortical stimulation in awake patients undergoing brain surgery Foerster demonstrated that stimulation of the intraparietal sulcus elicited full body rotations in space (Foerster, 1936), whereas a few years later stimulation of the superior temporal gyrus in patients operated on for focal epilepsy was associated with the sensations of “swinging, spinning,” “sinking feeling” and “head jumping up and down” (Penfield and Kristiansen, 1951; Penfield and Jasper, 1954; Penfield, 1957)

**FIGURE 1:**
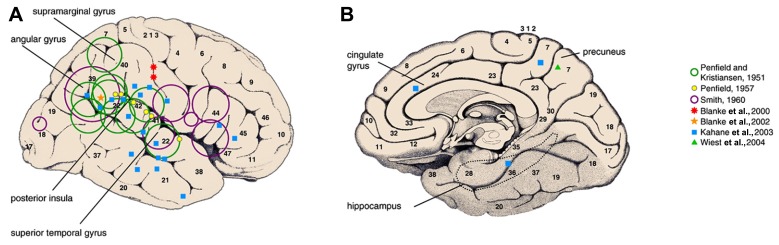
**Vestibular areas defined from direct electric cortical stimulations in epileptic patients.** Green and purple open circles represent the location of epileptogenic lesions responsible for vestibular sensations. Filled symbols represent the site at which focal electrical stimulation of the cortex evoked vestibular illusions in awake epileptic patients(reproduced from Lopez and Blanke, 2011).

More recently a new insight has been gained from a retrospective systematic study of intracranial electrical stimulation using depth electrodes in 44 refractory epilepsy patients (Kahane et al., 2003). It reported a wide distribution of anatomical sites from which vestibular sensations were electrically induced though confirmed that most sites were in the temporal and parietal areas. The authors suggested the presence of a human temporo-peri-Sylvian vestibular cortex (TPSVC), a possible equivalent to the monkey’s polysensory PIVC, but involving the insula less as stimulation of the insula infrequently evoked conscious vestibular sensations (Isnard et al., 2004). Stimulation of the parietal lobe more posteriorly, in area 39 near the angular gyrus has elicited non-specific vestibular sensations (Blanke et al., 2002). The angular gyrus has been previously proposed as the “epicenter of the vestibulo-psychic area” on the basis of lesional studies in epileptic patients (Smith, 1960). The data obtained from human stimulation studies are summarized in **Figure 1**.

The authors of the large retrospective intracerebral stimulation study were able to propose anatomical correlates to certain types of vestibular sensations (Kahane et al., 2003). Pitch plane illusions were mainly elicited from the parietal operculum and yaw plane illusions from the temporal cortex. Translational linear illusions were more frequently elicited by stimulation of the mesial structures, in particular the mesial parietal cortex. In a separate case report, stimulation of the right paramedian precuneus also reproduced the epileptic linear self-motion perception (Wiest et al., 2004). Another study using subdural grids, induced rotational and swaying bidirectional vestibular sensations by stimulating two adjacent sites at the anterior part of the intraparietal sulcus (Blanke et al., 2000).

This could suggest a three-dimensional coding of spatial information according to anatomical site, and the increasing complexity of bidirectional vestibular sensations could reflect higher level processing such as shown with the progressive increase in complexity the hierarchical organization of the visual and auditory systems.

Although this data contributes considerably to the understanding of the vestibular system a caveat to the stimulation on epileptic cortices is the possibility of cortical plasticity associated with recurrent seizures. This could explain the inter-individual variability in the studies.

### FOCAL SEIZURES AND VESTIBULAR SYMPTOMS

As well as the cortical stimulation studies, data has been gained by the close study of the electroclinical characteristics of focal seizures associated with vestibular dysfunction. Early studies in the 20th century (Penfield and Kristiansen, 1951; Smith, 1960) localized focal epileptic discharges associated with vertigo to the superior temporal gyrus and the temporo-parietal cortex.

**FIGURE 2:**
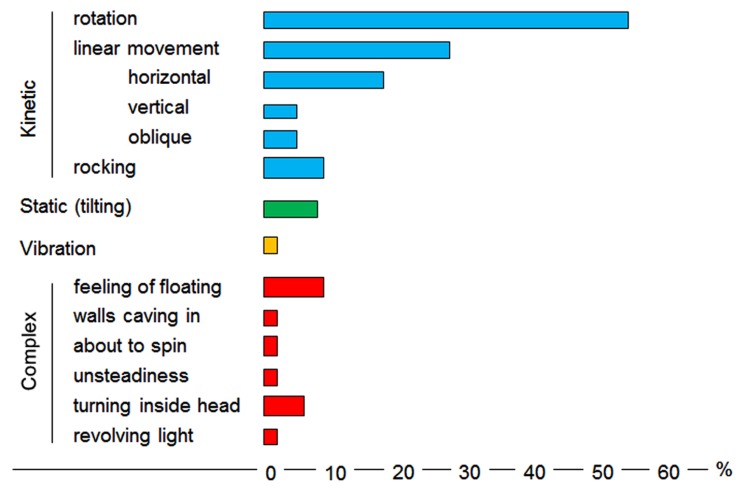
**Vestibular symptoms as reported in the Smith’s (1960) study (see comments in the text)**.

A particularly detailed clinical study of 120 patients revealed the commonest symptoms were the sense of rotational vertigo predominantly in the yaw and roll planes, linear translational illusions or a combination (Smith, 1960). More complex vestibular dysfunctions such as the sensation of floating, the anticipation of spinning and unsteadiness were also described supporting the theory of higher levels of organization (**Figure 2**).

More recently focal seizure activity associated with vestibular illusions of rotation involving of the temporo-parieto-occipital (TPO) junction has been demonstrated by stereotactic EEG (SEEG) recordings (Barba et al., 2007) and in a case using interictal SPECT and scalp EEG (Jaffe et al., 2006). Parietal seizures have been known to be often associated with vestibular sensations (Salanova et al., 1995), but in a recent SEEG study of the epileptogenic networks underlying parietal lobe seizures, vestibular sensations were associated with seizures arising from the superior parietal region (Bartolomei et al., 2011), in keeping with stimulation studies of intraparietal sulcus eliciting vestibular responses (Blanke et al., 2000). Parietal seizures could also elicit vestibular symptoms by interfering with areas associated with representation of the body in space (Blanke et al., 2000).

Vestibular symptoms are occasionally reported in other localisations, particularly in frontal lobe seizures (Lopez et al., 2010).

Focal seizures associated with vestibular sensations arising from these multiple distinct cortical areas is in keeping with current theories of a widespread vestibular multisensory cortical network (Lopez and Blanke, 2011).

## PURE “VESTIBULAR EPILEPSY”: A SEPARATE EPILEPTIC SYNDROME?

It is well recognized that vestibular symptoms commonly accompany more epileptic seizures, however, it is rare to have purely vestibular symptoms (Berkovic and Crompton, 2010). Despite the current perceived rarity of its existence attempts have been made since Gower to describe “pure” vestibular seizures (Alpers, 1960). In their report about “epileptic vertigo,” Jepsen and Pedersen (1956) reported two patients among 14 with pure vestibular seizures.

Two recent studies have attempted to more completely describe the diagnostic features of non-lesional epilepsies where the vestibular symptoms are the predominant features (Kogeorgos et al., 1981; Hewett et al., 2011; **Table 1**)

**Table 1:** Comparison between two series of patients with pure vestibular epilepsies.

	Kogeorgos et al. (1981)	Hewett et al. (2011)
Age at onset (mean, years)	25	26
Sex ratio M/F	1/1	2/1
Familial history	20%	28%
Febrile Sz	7%	-
Vestibular symptoms	100%	100%
Rotational	47%	78%
GTCS	23%	28%
Other clinical pictures	“Absences”	Neurocardiogenic syncopes, falls
Neuroimaging	N (CT-scan)	N (MRI)
EEG	Temporal posterior	TPO junction
Side predominance	Left	Right
Therapeutic response	Good	Good

*Sz, seizure; GTCS, generalized tonic-clonic seizures; N, normal; TPO, temporo-occipito-parietal.*

### CLINICAL FEATURES

Vestibular epilepsy is characterized by focal seizures with vestibular symptoms as either the sole or predominant feature. The vestibular symptoms can range from mild disequilibrium to frank vertigo in any plane of action (yaw, pitch, roll, linear), however, it is rare however to have purely vestibular symptoms. The most common accompanying symptoms are nausea or vomiting and tinnitus (Brandt, 2003; Hewett et al., 2011) but other well documented symptoms including ipsilateral and contralateral parasthesias, olfactory and gustatory hallucinations, depersonalisation, epigastric discomfort, anxiety and deja-vu which almost certainly reflect local seizure propagation (Kogeorgos et al., 1981).

Body or head and eye rotation with or without nystagmus is considered to be frequent in some descriptions, however this is not noted in the two published case series (Kogeorgos et al., 1981; Hewett et al., 2011).

A short period of altered consciousness has been considered a central characteristic of the epilepsy (Alpers, 1960). 50% of patients in series described by Kogeorgos et al. (1981) had brief “absences” with 23% having generalized tonic-clonic seizures (GTCS). 65% of patients had full loss of consciousness with a fall in the recent case series, though only 21% had GTCS (Hewett et al., 2011). In this series, a cardiogenic syncope (two had paroxysmal atrial fibrillation, and one with transient asystole during a focal seizure) or a vasovagal syncope (positive tilt table test) may have accounted for some of the loss of consciousness and fall. This association may just suggest epilepsy with concurrent cardiovascular disease, but in this young age group, it may suggest a link between the two conditions, particularly a predisposition for vasovagal hyperactivity.

The duration of seizures can be variable but is usually brief lasting a few seconds, though there are some patients reporting seizures lasting many minutes (Hewett et al., 2011). The symptoms are typically paroxysmal beginning suddenly and unless the seizure secondarily generalizes they usually discontinue abruptly. In cases of pure vestibular symptomatology these features can be particularly helpful in differentiating from other vertiginous diagnoses (see below; Alpers, 1960; Brandt, 2003).

### ELECTROENCEPHALOGRAPHY

A diagnosis will be heavily supported by positive EEG findings and lateral temporal epileptic foci are frequent (Alpers, 1960; Brandt, 2003).

In the series described by Kogeorgos et al. (1981) an abnormal interictal EEG was a major criterion for the diagnosis. 28/30 had a temporal or bitemporal abnormalities, in some associated with generalized seizure discharges. There was a left temporal emphasis in 50%, right in 25% and bitemporal in 20%. The other two had atypical generalized patterns.

**FIGURE 3:**
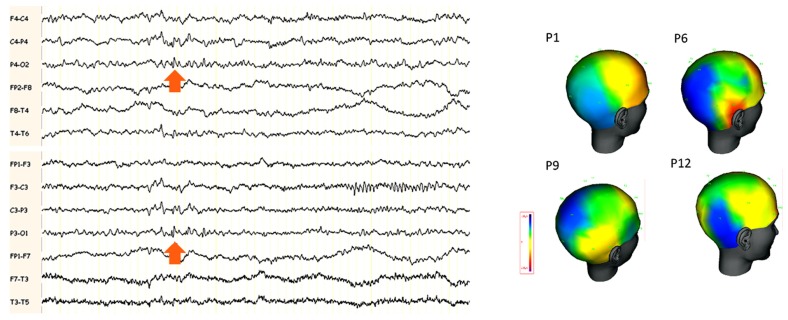
**(A)** Example of scalp EEG with interictal abnormalities in the posterior electrodes in a patient with “benign TPO junction epilepsy.” The arrows indicate small spikes and waves around the electrodes P3 and P4 bilaterally. **(B)** Amplitude cartography using coherence (deltamed-natus) software in four patients with benign TPO junction epilepsy (adapted from Hewett et al., 2011). One representative single interictal spike is shown. Note the negative distribution (in blue) over the TPO region, maximal in T6, P4 and O2 in the four patients.

In the series described by Hewett et al. (2011), the predominant features on interictal scalp EEG were abnormalities over the parietal or TPO areas (**Figure 3**). Many of these activities are close in their morphology and topography to lambda waves and are therefore difficult to distinguish from physiological activity. A right side predominance was also found in this series.

### FAMILY HISTORY

A family history of epilepsy was elicited in 20% (Kogeorgos et al., 1981) and 29% (Hewett et al., 2011) had some form of family history but none with first-degree relative. A genetic link is therefore possible in these cases and a family history of epilepsy is a helpful feature in differentiating from the other vestibular syndromes.

### MANAGEMENT

Vestibular seizures are considered to respond well to anti-epileptic medication (Brandt, 2003). In the oldest series, over half of the patients had complete remission with phenytoin or carbamazepine with a considerable reduction in the frequency and severity of attacks with the remainder who complied (Kogeorgos et al., 1981). All the patients that complied to treatment (12/14) in the other case series were seizure free at time of publication on either mono or dual therapy with more modern medication (Hewett et al., 2011).

### DIFFERENTIAL DIAGNOSIS

#### Vestibular syndromes

The short duration of the symptoms and abruptness of recovery would exclude peripheral vestibular syndromes such as Meniere’s disease or vestibular neuritis.

Basilar/vestibular migraine is considered to be the most common cause of spontaneous episodic vertigo. The duration varies from seconds to days, usually lasting minutes to hours, and they mostly occur independently of headaches (Bisdorff, 2011). Interestingly the anticonvulsant Lamotrigine seems to be more effective for the vertigo attacks than the headaches (Cha, 2010) raising the possibility of misdiagnosis or overlap between vestibular migraine and epilepsy.

At least six primary episodic ataxia (EA) syndromes have been described (Jen et al., 2007), of which EA1 and EA3 could mimic vestibular epilepsy most closely.

EA1 presents with brief episodes of ataxia lasting seconds to minutes and phenotypic variants combine partial epilepsy. EA3 presents with episodic vertigo, tinnitus and ataxia typically lasting minutes. Interestingly, there is clear overlap in clinical features between EA3 and migraine-associated vertigo.

Transient ischaemic event in vertebrobasilar territory, and the rare paroxysmal brainstem attacks with ataxia/dysarthria in MS can all mimic vestibular seizures due to their brief duration, but associated brainstem dysfunction aids in their differentiation (Brandt, 2003; Dieterich, 2007). Variability of the symptoms and accompanying functional symptomatology can support diagnosis of psychogenic cause (Cherchi, 2011) and phobic postural vertigo is commonly associated with an obsessive personality (Brandt, 1996).

#### Other seizures

A vestibular seizure is not difficult to distinguish from vertiginous syndromes if accompanied by other epileptic features, though other epileptic seizures need to be considered. Limbic seizures arising from the mesial temporal lobe present with prominent psychic (perceptual illusions, mnemonic or emotional) or autonomic features that can either be associated with vertiginous syndromes or could be mistaken by the patient to be vestibular symptoms (Maillard et al., 2004). Rotatory seizures (volvular epilepsy or circling epilepsy) are characterized by paroxysmal repetitive walking in small circles, the direction of rotation usually contraversive to the epileptic focus and preceded by versive movements of the head and body in the same direction (Brandt, 2003).

#### Vestibulogenic seizures

Vestibular epilepsy should not be confused with the distinct classical and historically defined condition of “vestibulogenic epilepsy.” This is a variety of sensory-evoked epilepsy caused by an inner ear disorder or provoked by peripheral labyrinthine stimulation (Brandt, 2003). These are considered to be example of a reflex epilepsy. In extremely rare cases seizures with only vestibular manifestations and EEG discharges localized into the temporo-parietal region are triggered by vestibular stimuli (Gizzi and Diamond, 2005). However, this mode of provocation is probably not specific in the majority of cases (Karbowski, 1989).

#### Presyncopal symptoms

A major differential to consider and separate from epileptic vestibular symptoms are presyncopal symptoms.

Patients describing genuine vaso-vagal or cardiogenic pre-syncopal symptoms will commonly use the term dizziness and may confuse these with vestibular symptoms (Wieling et al., 2009). Strict clarification is necessary. This is challenging as the term dizziness has also been attributed to many different sensations that can be associated with vestibular seizures, e.g. apprehension, mental confusion, pressure in the head, tinnitus, gastrointestinal awareness, nausea, “auditory disturbance,” and blindness. Furthermore some patients, as in the most recent series, do have true syncopal manifestations associated with vestibular epilepsy (Hewett et al., 2011).

## CONCLUSION: AN UNDER RECOGNIZED FORM OF EPILEPSY?

Vestibular epilepsy can offer difficulty in recognition and is still perceived as extremely rare (Dieterich, 2007; Crompton and Berkovic, 2009) unless there are clear epileptic features, diagnosis is often delayed. In the most recent case series, the average delay in diagnosis following onset of symptoms was 4 years (Hewett et al., 2011) and although 11/14 patients had other features of seizure activity, a majority (Brandt, 2003) were initially seen by otorhinolaryngologist or cardiologist before review by a neurologist.

When diagnosed, the vestibular seizures have been regarded as a heterogenous group of partial seizures. However, this recent case series of patients describe a series of adolescents and adults who share defining electroclinical characteristics of a non-lesional pharmacoresponsive epilepsy manifesting as prominent vestibular disturbances. Many of these characteristics are shared by the larger case series described 30 years earlier (Kogeorgos et al., 1981).

Given the relatively young onset, the family history, and relatively “benign” nature of these epilepsies, we propose that they may represent more than just a number of heterogeneous group of cryptogenic partial epilepsies. Although further characterisation is required, this raises the possibility of a form of idiopathic epilepsy. We recently proposed the term “benign temporo-parieto-occipital junction epilepsy with vestibular disturbance” to characterize this condition (Hewett et al., 2011).

## Conflict of Interest Statement

The authors declare that the research was conducted in the absence of any commercial or financial relationships that could be construed as a potential conflict of interest.
